# Potential Cardioprotective Effect of a GRK5 Inhibitor Against NF-κB-Mediated Inflammation in an Animal Model of Isoproterenol-Induced Myocardial Infarction

**DOI:** 10.3390/ijms27010053

**Published:** 2025-12-20

**Authors:** Asma S. Alonazi, Anfal F. Bin Dayel, Bashayer A. Alkhathlan, Lulu M. Alkaff, Ahad T. Alrashed, Reema A. Bin Klaib, Doaa M. Elnagar, Maha A. Alamin, Rehab A. Ali, Alaa Alnoor Alameen, Nouf M. Alrasheed

**Affiliations:** 1 Department of Pharmacology and Toxicology, College of Pharmacy, King Saud University, Riyadh 11451, Saudi Arabia; 2 Zoology Department, College of Science, King Saud University, Riyadh 11451, Saudi Arabia

**Keywords:** GRK5, Amlexanox, NF-κB, inflammation, myocardial infarction

## Abstract

Myocardial infarction (MI) is a pathological condition associated with various cardiovascular diseases and leads to heart failure. Nuclear factor-kappa B (NF-κB) is upregulated in the infarcted heart. G protein-coupled receptor kinase 5 (GRK5) also plays a complex role in both tissue repair and maladaptive hypertrophy in cardiovascular diseases; however, its effect on NF-κB-mediated inflammation has not yet been elucidated. Thus, this study aims to investigate the effects of Amlexanox (AMX), a potential GRK5 inhibitor, in an animal model of MI by assessing its impact on GRK5-mediated NF-κB/inflammatory processes. Thirty-two male mice were randomly allocated into four groups: control, MI, (MI treated with vehicle (MI + V), and MI + AMX (AMX: 2.5 mg/100 g/day). MI was induced using ISO on days 21 and 22. The cardioprotective impacts of Amlexanox were verified by evaluating cardiac injury, inflammatory biomarker concentrations, and histopathological alterations in cardiomyocytes. MI induction was confirmed by increases in heart weight/body weight ratio (HW/BW) (*p* < 0.001), troponin (*p* < 0.001), creatine kinase (*p* < 0.001), and LDH (*p* < 0.001). Treatment with AMX resulted in a significant reduction in cardiac injury biomarkers (*p* < 0.001) and IL-6 (*p* < 0.05). The protein level of NF-κB(p65) and NF-κB(p105) was significantly increased in cardiac myocytes of the MI group. Treatment with AMX led to a significant decrease in NF-κB(p65) and (p105) expression (*p* < 0.01 and *p* < 0.001, respectively), and GRK5 and MEF2α protein levels were also upregulated. In conclusion, AMX shows potential cardioprotective effects by modulating the GRK5/MEF2-mediated NF-κB inflammatory signaling pathway.

## 1. Introduction

Cardiovascular diseases (CVDs) are considered one of the most prominent causes of death, disability, and increasing health care costs. The growing prevalence of such disease is associated with various risk factors, such as hypertension, smoking, diabetes, dyslipidemia, poor physical activity, and diet [1]. Recent studies involving Gulf Council Countries, including Saudi Arabia, have indicated that CVDs cause more than 45% of fatalities in Saudi Arabia and are considered a significant health burden. A large incidence of stroke at a young age (≤45 years old) has also been documented in Gulf Countries in several studies, with 9.8% to 25% of those affected by stroke being young individuals. According to the world health organization (WHO), CVDs account for 37% of all noncommunicable diseases in Saudi Arabia, making it the most prevalent noncommunicable disease [1,2]. Several recent studies have also indicated that CVDs are among the most frequently diagnosed diseases in multimorbidity cases, alongside hypertension, diabetes, obesity, and coronary heart disease [3].

An important feature of CVDs is myocardial infarction (MI), which is described as a decline in blood-oxygen supply to part of the myocardium or its total cessation. The pathophysiological changes associated with MI begin with a significant and protracted imbalance between the actual and required cardiac O_2_ supply. The most common cause of this is luminal thrombus superimposed on occlusive coronary atherosclerosis, resulting in pathological changes, including endothelial dysfunction and inflammation [4].

Inflammation caused by ischemia and cellular death is considered to be a critically important factor in the progression of MI. The process of inflammation involves a diverse group of immune cells that produce cytokines/mediators, which play a role in the continuous damage and recruitment of more immune cells to the area of inflammation [5]. This process involves humoral (complement system, reactive oxygen species (ROS), cytokine cascade) and cellular (neutrophil rolling/infiltration, chemokines)-mediated inflammatory responses. Several cytokines are released during the inflammation process, for example, nuclear factor kappa B (NF-κB) as well as pro-inflammatory mediators, tumor necrosis factor-α (TNF-α), interleukin-6 (IL-6), and IL-1 [6]. Early pro-inflammatory cytokines like TNF-α, IL-6, and IL-1β are elevated during the initial response to MI. In the beginning, the acute release of pro-inflammatory cytokines regulates survival or cellular apoptosis in the infarcted region. However, if the production of these cytokines is continuously increased, this will cause interstitial fibrosis and collagen accumulation in the non-infarcted region, which will result in ventricular dysfunction [7]. The NF-κB family, consisting of cytokines released during inflammation, is a family of five-membered transcriptional factor proteins sharing the same Rel homology domain. They mediate and control many processes, including inflammation. Normally, they exist in the cytoplasm as a complex with an inhibitor protein called Inhibitor of κB (IκB). It has been shown that myocardial ischemia and reperfusion are the primary causes of the activation of NF-κB. Upon activation, there are two main signaling pathways that are crucial for controlling the inflammatory and immunological responses of NF-κB: canonical and non-canonical (alternative) pathways. Both are involved in the process of NF-κB activation but have different signaling mechanisms. Upon stimulation of the canonical pathway by different receptors, such as pattern recognition receptors (PRRs), the TNF receptor (TNFR), and cytokine receptors, the NF-κB and IκB complex dissociates and degrades via IκB serine residue phosphorylation, caused by IκB kinase (IKK). The free NF-κB is then translocated into the nucleus, inducing gene expression. The primary signaling molecule for the non-canonical pathway is NF-κB-inducing kinase (NIK), and when this pathway is stimulated, NF-κB2 precursor protein (p100) will be phosphorylated and processed into p52, which in turn will translocate into the nucleus, also triggering gene expression [7,8].

G protein-coupled receptor kinase 5 (GRK5) is one of the GRK group and has been linked to several human diseases, such as heart failure, hypertension, cancer, diabetes, and Alzheimer’s disease [9]. The GRK5 protein has between 500 and 700 amino acids and has some characteristics in common with other GRK superfamily members. A C-terminal region of varying length (between 105 and 230 residues) surrounds the core catalytic domain of GRK5, which has about 270 residues. GRK5 localizes in the plasma membrane through a membrane-binding domain in the C-terminal RH domain. The N-terminal domain, a nuclear localization sequence (NLS) that distinguishes GRK5 from the other GRKs, appears to be important for receptor recognition and intracellular membrane localization. The capacity of GRK5 to translocate to the nucleus and engage in its non-canonical activity is due to this NLS [10].

Previous reports show that GRK5 is upregulated in CVDs such as heart failure [11]. The nuclear translocation of GRK5 is regulated by the interaction of GRK5 and calmodulin, which occurs after activation of the membrane GPCR, leading to its nuclear translocation and subsequently inducing this pathology [12,13]. There is a nuclear localization sequence (NLS) on GRK5 that is capable of binding DNA. Studies have demonstrated that when GRK5 enters the nucleus, it begins to function as a histone deacetylase (HDAC) kinase [14]. HDAC is phosphorylated, which results in its nuclear export and the subsequent de-repression of MEF2, which activates the transcription of genes related to hypertrophy [11]. Another critical regulator of the hypertrophic gene is the nuclear factor of activated T-cells (NFAT), which is activated in GRK5-mediated pathological cardiac hypertrophy, as GRK5 promotes NFAT-mediated hypertrophic gene transcription. In addition, in genetically modified mice, GRK5 overexpression resulted in the loss of NFATc3, which leads to protection against increased hypertrophy and the early development of HF, as observed in transverse aortic constriction [15].

Many studies have analyzed the role of GRK5 in MI. Here, we will focus on two of these studies. The first concentrated on the protective role of GRK5 in MI, while the other focused on its harmful effects. The first study highlights that one of the mechanisms involved in tissue repair after cardiac damage caused by MI is the differentiation of myofibroblasts from resident fibroblasts, leading to the production of collagen to repair the damaged area. GRK5 is extensively expressed in post-MI mouse hearts, as well as in heart myofibroblasts. GRK5-mediated activation of NF-κB leads to the expression of fibrosis-related genes, thereby decreasing cardiac damage. In contrast, GRK5 knock-out mice have shown increased mortality due to suppression of inflammation and fibrosis [16]. On the other hand, another study showed that GRK5 causes maladaptive cardiac hypertrophy and is increased in failing human myocardium. The exact function of GRK5 in myocardial infarction is still unclear [17]. One published study assessed the possible vital role of GRK5 in post-MI using cardiomyocyte-specific GRK5 knockout mice (GRK5cKO) and cardiomyocyte-specific GRK5-overexpressing transgenic mice (TgGRK5) [17]. This study showed that TgGRK5 post-MI mice demonstrated reduced cardiac functionality, increased left ventricular dimensions, and a lower survival rate. Moreover, immune regulators were created, eventually leading to chronic cardiac inflammation and increased and persistent leukocyte recruitment into the damaged heart. At 4 days and 8 weeks post-MI, higher levels of neutrophils, macrophages, and T lymphocytes, as well as pro-inflammatory neutrophils and macrophages, were observed. As opposed to WT post-MI mice, GRK5cKO animals showed decreased early immune cell recruitment (mostly monocytes) to the heart, enhanced contractility, and decreased mortality [17]. These data all show the important role of GRK5 in cardiac myocytes post-MI. However, there are no published studies that investigated GRK5 kinase activity in MI-induced inflammatory processes. Amlexanox has been shown to exert an inhibitory function on GRK5 kinase activity [18], even though its effect on GRK5 in cardiac myocytes during MI has not been investigated. Therefore, this study aims to determine whether pharmacologic inhibition of GRK5 may possibly affect the post-MI inflammatory progression.

## 2. Results

### 2.1. Effect of Amlexanox on ISO-Induced Cardiac Injury, Inflammatory Biomarkers, and Morphology

**Figure 1:**
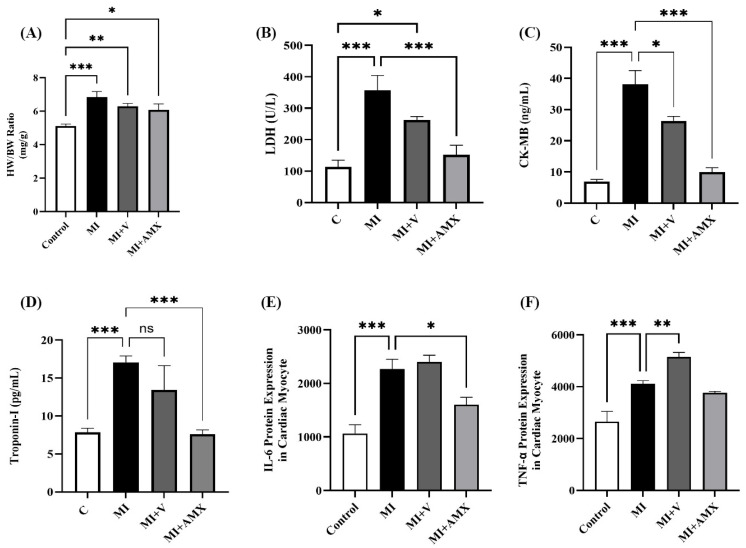
Effects of Amlexanox on MI-induced cardiac injury biomarkers and inflammatory cytokines. The HW/BW ratio as an indicator of cardiac remodeling after MI (**A**). Serum levels of cardiac injury biomarkers LDH (**B**), CK-MB (**C**), and Tn-I (**D**). Serum levels of IL-6 (**E**) and TNF-α as an indicator of inflammation (**F**). All data are expressed as mean ± SEM (n = 6 mice per group). Differences between groups were revealed using one-way ANOVA followed by Tukey’s post hoc test. Statistically significant variations are presented as * *p* < 0.05, ** *p* < 0.01, *** *p* < 0.001, ns: non-significant. Abbreviations: C: control; MI: untreated myocardial infarction; MI + V: myocardial infarction treated with vehicle; MI + AMX: myocardial infarction treated with Amlexanox.

**Figure 2:**
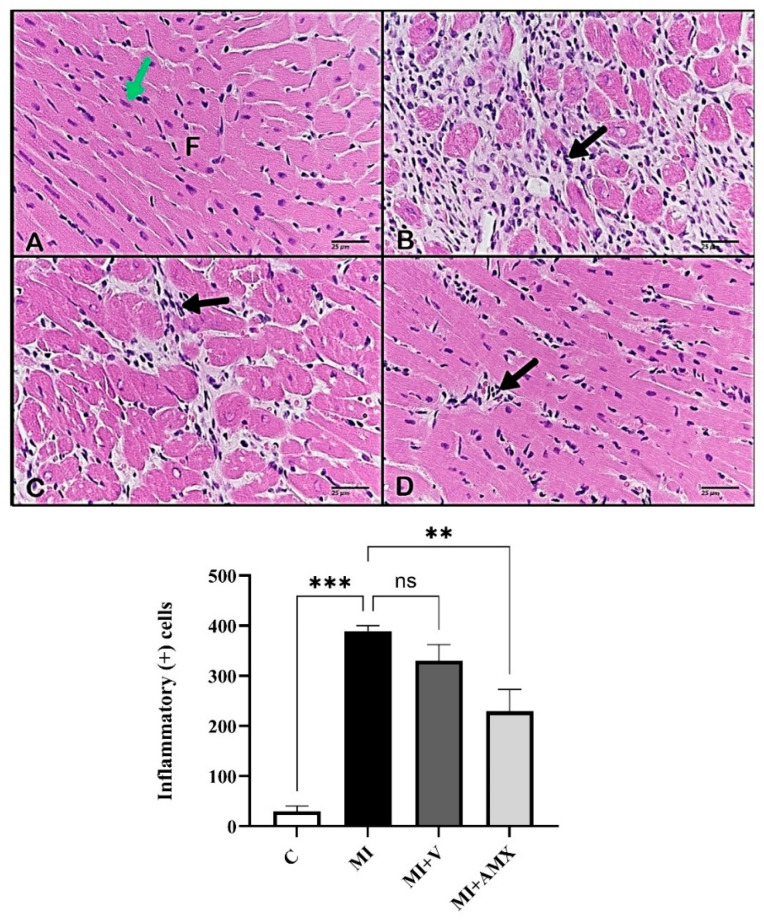
Effects of Amlexanox on MI-induced cardiac morphology and inflammatory cells. Photomicrographs of cardiac muscles (H&E): (**A**) Control cardiac muscles exhibiting normal features. (**B**) Cardiac muscles of mice from the MI group, revealing invasion of inflammatory cells. (**C**) Cardiac muscles of animals treated with vehicle (MI + V), showing marked inflammation. (**D**) Cardiac muscles of mice from the AMX-treated group, showing lower inflammatory cell accumulation (40×). All data are expressed as mean ± SEM (n = 3 mice per group). Differences between groups were revealed using one-way ANOVA followed by Tukey’s post hoc test. Statistically significant variations are shown as ** *p* < 0.01, *** *p* < 0.001, ns: non-significant. Abbreviations: C: control; MI: untreated myocardial infarction; MI + V: myocardial infarction treated with vehicle; MI + AMX: myocardial infarction treated with Amlexanox. Cardiac fibers (F), central nucleus (green arrow), inflammatory cells (black arrows).

To understand the effect of Amlexanox in cardiac remodeling, the HW/BW ratio, cardiac injury, and inflammatory cytokines were evaluated. The HW/BW ratio was significantly enhanced in infarcted hearts (*p* = 0.0002); (*p* = 0.093); and (*p* = 0.043) for MI, MI + V, and MI + AMX, respectively (Figure 1A). Treatment with either the vehicle or Amlexanox did not affect HW/BW compared to the untreated group. Cardiac injury biomarkers such as Troponin I, CK-MB, and LDH were also evaluated, with the cardiac LDH serum level showing a significant increase in the MI untreated group. Treatment with Amlexanox markedly reduced the LDH serum level compared to the untreated group (*p* < 0.001) (Figure 1B). Similarly, serum CK-MB (Figure 1C) and Troponin I (Figure 1D) were significantly enhanced in the infarcted heart (*p* < 0.001). Treatment with Amlexanox significantly decreased serum CK-MB and Troponin I compared to the untreated group (*p* < 0.001). Tissue IL-6 and TNF-α levels were also evaluated as indicators of inflammation. As shown in Figure 1E, IL-6 showed a significant increase in the MI untreated group (*p* = 0.0005), while treatment with Amlexanox markedly reduced IL-6 serum levels compared to this group (*p* = 0.0495). Similarly, TNF-α levels (Figure 1F) were significantly enhanced in infarcted hearts (*p* = 0.0002), but treatment with Amlexanox resulted in a non-significant reduction in these levels compared to the untreated group. Figure 2 provides further insights from H&E staining experiments. Control cardiac muscles exhibited normal myocardial fibers with abundant central nuclei (Figure 2A). However, mice in the MI group exhibited severe inflammation between cardiac muscles (Figure 2B); similarly, the cardiac muscles of MI animals treated with vehicle exhibited marked inflammation (Figure 2C). MI animals treated with Amlexanox exhibited a marked improvement in cardiac muscles, caused by lower inflammatory cell accumulation (Figure 2D).

### 2.2. Effect of Amlexanox on NF-κB Gene and Protein Expression

**Figure 3:**
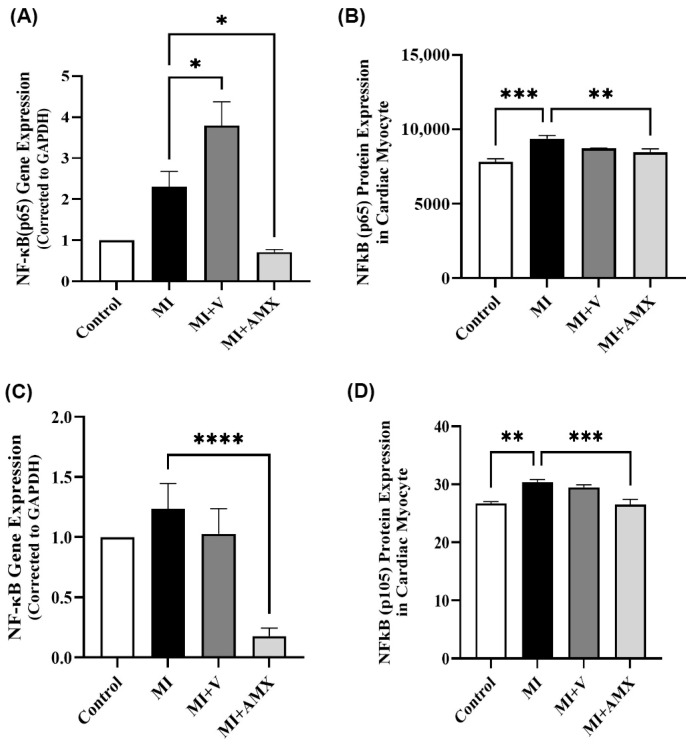
Amlexanox reduced *NF-κB* gene and protein expression. *NF-κB*(*p65*) gene (**A**) and protein (**B**) expression in myocardium tissues. *NF-κB* gene (**C**) and protein (**D**) expression in myocardium. All data are expressed as mean ± SEM (n = 4 independent mice per group). Differences between groups were verified using one-way ANOVA followed by Tukey’s post hoc test. Statistically significant varies are indicated as * *p* < 0.05, ** *p* < 0.01, *** *p* < 0.001, **** *p* < 0.0001. Abbreviations: C: control; MI: untreated myocardial infarction; MI + V: myocardial infarction treated with vehicle; MI + AMX: myocardial infarction treated with Amlexanox. NF-κB: Nuclear factor kappa-light-chain-enhancer of activated B cells. Apparent differences without statistical markers indicate non-significant variation following post hoc correction despite numerical changes.

NF-κB(p65) subunit protein levels were increased in the myocardium of infarcted mice compared to control mice (*p* = 0.0001). Treatment with Amlexanox reduced *NF-κB*(*p65*) subunit gene expression and protein levels compared to the MI-untreated group (*p* < 0.01 and *p* = 0.008, respectively) (Figure 3A,B). Similarly, NF-κB(p105) subunit protein levels were increased in the myocardium of infarcted mice compared to control mice (*p* = 0.003). Amlexanox treatment reduced NF-κB(p105) subunit levels compared to the MI-untreated group (*p* < 0.01 and *p* = 0.0007, respectively) (Figure 3C,D).

### 2.3. Amlexanox Upregulates GRK5 and MEF2α in ISO-Induced Myocardial Infarction

**Figure 4:**
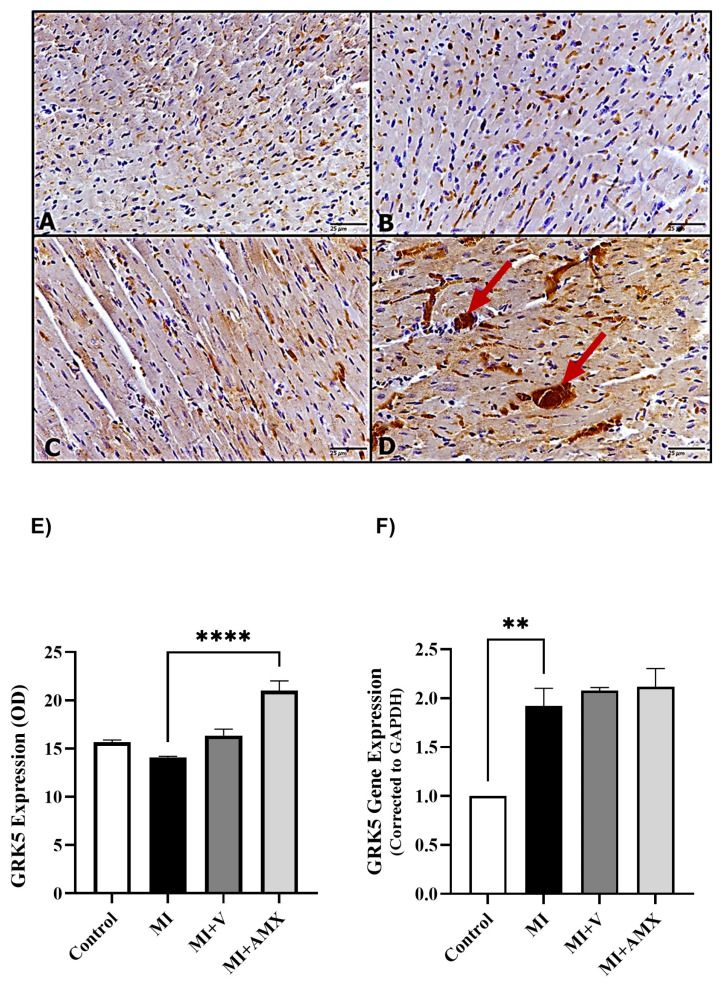
Effects of Amlexanox on GRK5 protein level and *GRK5* gene expression. Photomicrographs (**upper panel**) showing normal GRK5 expression in the control group (**A**), increased expression in the MI (untreated) group (**B**), and MI-treated-with-vehicle group (**C**). Upregulated GRK5 expression was observed in the MI + AMX group (**D**), as indicated (red arrows) (magnification 40×). Semi-quantification results for GRK5 immunoreactivity (lower panel—(**E**)) (using ImageJ software version 1.53t). Expression of *GRK5* gene in myocardium tissue using RT-PCR (**F**). All data are expressed as mean ± SEM (n = 3 different analyses per group; multiple sections and randomly selected fields were analyzed per animal for immunohistochemical study). Differences between groups were revealed using one-way ANOVA followed by Tukey’s post hoc test. Statistically significant differences are shown by ** *p* < 0.01 and **** *p* < 0.0001. Abbreviations: C: control; MI: untreated myocardial infarction; MI + V: myocardial infarction treated with vehicle; MI + AMX: myocardial infarction treated with Amlexanox. Lack of statistical significance between some groups reflects basal GRK5 expression and biological variability rather than absence of treatment effect.

**Figure 5:**
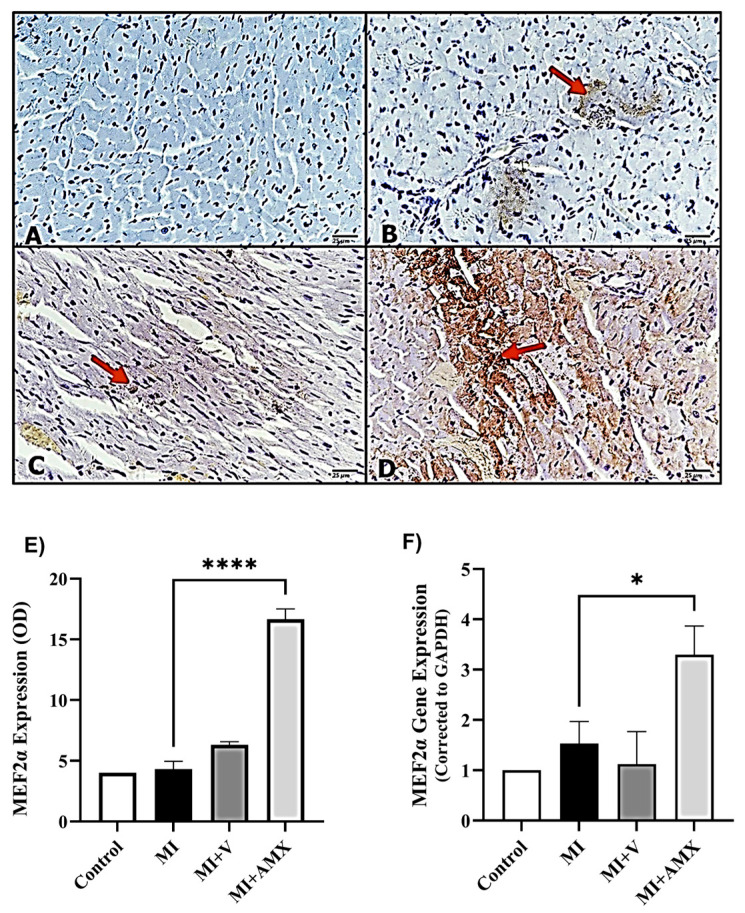
Effects of Amlexanox on MEF2α protein and gene expression. Photomicrographs (**upper panel**) showing normal MEF2α expression in the control group (**A**), increased expression in the MI (untreated) group (**B**), and the MI-treated-with-vehicle group (**C**). Upregulated MEF2α was observed in the MI + AMX group (**D**), as indicated (red arrows) (magnification 40×) (scale 25 µm). Semi-quantification results for MEF2α immunoreactivity (lower panel—(**E**)) (using ImageJ software). Expression of MEF2α in myocardium tissue using RT-PCR (**F**). All data are expressed as mean ± SEM (n = 3 different analyses per group). Differences between groups were revealed using one-way ANOVA followed by Tukey’s post hoc test. Statistically significant differences are shown by * *p* < 0.05 and **** *p* < 0.0001. Abbreviations: C: control; MI: untreated myocardial infarction; MI + V: myocardial infarction treated with vehicle; MI + AMX: myocardial infarction treated with Amlexanox. Significance between MI and MI + AMX indicates normalization toward control levels; comparisons not marked were not statistically significant after post hoc testing.

Immunohistochemistry of cardiac muscles for GRK5 expression revealed weak immunostaining of GRK5 in mice with injured myocardium (MI) (Figure 4B). Muscles of Amlexanox-treated animals exhibited more intense expression than the other groups (Figure 4D). Moreover, as shown in Figure 5, following immunohistochemical staining of cardiac myocytes against MEF2, no immunostaining was observed in the control group. At the same time, MI untreated mice showed weak MEF2α expression and the MI-vehicle-treated mice (MI + V) exhibited higher MEF2α expression, while the (MI-AMX)-treated mice displayed more intense MEF2α expression compared to the previous groups. Similarly, the expression of MEF2α was significantly increased in the MI-AMX-treated group compared to the MI-untreated group (*p* < 0.05) (Figure 5F).

All statistical analyses for *NF-κB*, *GRK5*, and *MEF2α* gene and their protein expression re-checked and confirmed using one-way ANOVA followed by Tukey`s post hoc test. Apparent numerical differences that did not reach statistical significance reflect inter-individual biological variability and overlapping dispersion between groups within the experimental sample size. Therefore, only post hoc-corrected statistically significant comparisons are indicated, and Amlexanox effects are interpreted as normalization toward control levels rather than indication of supraphysiological expression.

## 3. Discussion

GRK5 is a crucial regulator of β1-adrenergic receptors and their related signaling cascades [19]. Several studies have investigated the role of GRK5 in cardiovascular diseases [9,11], including, importantly, its effect on MI [16,17]. Amlexanox has been shown to exert an inhibitory function on GRK5 [18]. In the current study, we displayed the cardio-protection influence of Amlexanox, a GRK5 inhibitor, in an animal model of MI, particularly focusing on its inhibitory effect on GRK5 modulation of NF-κB-mediated inflammatory effects.

Myocardial infarction is associated with inflammation, which contributes to pathological cardiac remodeling [20,21]. We used isoproterenol as a noninvasive method for inducing pathological myocardium damage [22,23], and various cardiac biomarkers were applied as indicators of myocardial injury, including the HW/BW ratio, troponin-I (Tn-I), LDH, and CK-MB. ISO-treated mice confirmed an apparent increase in the HW/BW ratio and serum cardiac enzymes, including Tn-I and CK-MB. Circulating Tn-I is a highly specific biomarker of myocardial injury, and even a minimal elevation in its levels reflects acute cardiomyocyte damage [24,25]. In addition, marked CK-MB elevation has been consistently reported in experimental models of ISO-induced myocardial infarction [26,27,28]. Consistent with these reports, our experiments showed that Tn-I and CK-MB levels were significantly increased in the MI group compared to the control group, confirming successful MI induction. Our findings demonstrate that Amlexanox treatment attenuates ISO-induced myocardial damage. This is evident by the significant reduction in cardiac injury biomarkers observed in histopathological analysis. In this study, Amlexanox treatment did not reduce the HW/BW ratio compared to the MI group, but it did remain significantly higher than that in the control group. These findings are consistent with those reported by Adizak et al. (2021) [29]. However, another study demonstrated a significant reduction in the HW/BW ratio following Amlexanox treatment compared to the isoproterenol-induced MI group [30]. This discrepancy may be due to differences in ISO administration protocols, particularly the duration and dosing schedule, across studies. Importantly, treatment with Amlexanox resulted in a significant reduction in Tn-I levels compared to the MI group. This aligns with a previous report that observed troponin levels were significantly reduced compared to the vehicle group [29]. Amlexanox treatment also markedly reduced CK-MB compared to the MI group, suggesting attenuation of myocardial injury. Although studies directly investigating the effect of Amlexanox on CK-MB are lacking, our results support its potential cardioprotective role in a mouse model of ISO-induced MI.

NF-κB is considered a major transcription factor associated with MI and is involved in the release of pro-inflammatory factors that participate in various signaling pathways in the MI pathophysiology. It regulates the production of pro-inflammatory mediators and contributes to cardiomyocyte apoptosis [31]. Recent studies have shown that NF-κB inhibition has a protective role in MI injury [31]. In our study, NF-κB(p105) and (p65) subunit levels were significantly elevated in MI mice. This finding is in agreement with a previous report showing upregulation of NF-κB in ISO-induced cardiac hypertrophy models [32]. Importantly, our results demonstrated a significant reduction in NF-κB(p105) and (p65) levels in Amlexanox-treated mice. To our knowledge, no previous study has directly investigated the effect of Amlexanox on NF-κB signaling in myocardial infarction models. However, studies on cardiac remodeling models have reported that Amlexanox attenuates NF-κB activation, primarily through suppression of inflammatory signaling pathways and inhibition of (p65) phosphorylation. In contrast to our findings, Adzika et al. (2021) reported that persistent NF-κB activation occurred in Amlexanox-treated groups despite treatment [29]. This may be attributed to the different experimental designs between the two experiments. Another study supports our finding that Amlexanox downregulates NF-κB mechanistically [33]. These findings suggest that Amlexanox’s potential mechanism in MI settings may be through cardioprotective effects by selectively downregulating NF-κB subunit activity. The observed differences in NF-kB gene and protein expression likely reflect dynamic regulation of inflammatory signaling following myocardial injury and pharmacological modulation of GRK5, which may contribute to variability in expression patterns among experimental groups.

TNF-α is a key early pro-inflammatory cytokine after myocardial injury and contributes to cardiomyocyte dysfunction, cell death, and adverse remodeling; therefore, it is commonly used as a marker of post-MI inflammation [34]. Evaluating myocardial TNF-α allows direct assessment of ISO-induced myocardial injurious inflammation in an animal model of MI. Using this rationale, multiple ISO-induced cardiac injury studies in mice have reported significant increases in myocardial or plasma TNF-α following ISO administration. For example, Obeidat et al. demonstrated elevated inflammatory markers, including TNF-α and IL-6, in an ISO-induced mouse model, consistent with our finding that MI significantly increases IL-6 and TNF-α levels in the myocardium tissue [35]. A previous study investigated the effects of Amlexanox in an ISO-induced cardiomyopathy mouse model, showing that Amlexanox alone produced only partial attenuation of inflammatory markers, whereas its combination with forskolin more effectively reduced ISO-induced myocardial inflammation and remodeling [29]. Another study reported that Amlexanox suppresses pro-inflammatory mediators in LPS-stimulated microglial cells and activates macrophages [33,36], confirming that Amlexanox exerts an anti-inflammatory effect [37]. In line with these findings, current results demonstrated that Amlexanox significantly reduced IL-6 and partially reduced TNF-α levels, confirming its effect on NF-κB-pro-inflammatory pathway activation.

A previous study reported that Amlexanox exerts an inhibitory function on GRK5 [18]. This study focused on its inhibitory influence on GRK5 modulation of NF-κB-mediated inflammatory consequences. A previous study reported that GRK5 expression was increased in a mouse model of MI, particularly in cardiac fibroblasts and myofibroblasts, and that GRK5 promoted the expression of inflammation-related genes through NK-κB activation, leading to an increase in the expression of fibrotic genes [16]. GRK5 has been shown to enhance MEF2 activity by translocating to the nucleus, where it phosphorylates histone deacetylase-5 (HDAC5), thereby promoting MEF2α-mediated hypertrophic gene expression [15].

Importantly, a recently published paper reported that *MEF2*-regulated genes inhibit cardiomyocyte inflammation [38], highlighting a possible link between MEF2 and NF-κB-mediated inflammation. Our findings show that Amlexanox caused an upregulation in GRK5 expression in an MI mouse model and consequently upregulated MEF2α expression, suggesting that upregulating GRK5/MEF2α has a possible role in the downregulation of NF-κB-mediated inflammation. This is consistent with previous findings that show that Amlexanox abolishes the GRK5-mediated induction of inflammatory responses [29].

Although Amlexanox is classified as a GRK5 inhibitor, its inhibitory effect primarily targets GRK5 kinase activity rather than suppressing GRK5 gene or protein expression [18]. Accordingly, the increased GRK5 expression observed in the MI + AMX group does not contradict its inhibitory role. GRK5 expression is dynamically regulated following myocardial injury and may increase as a part of an adaptive or compensatory response during the post-infarction phase. Emerging evidence suggests that GRK5 exerts context-dependent roles in the heart, displaying either maladaptive or cardioprotective effects depending on disease stage, cellular localization, and signaling environment [11,16,17]. Notably, GRK5 has been shown to promote tissue repair and survival following myocardial infarction through regulation of inflammatory and transcriptional programs [16]. Importantly, GRK5 signaling output depends on its kinase activity and subcellular localization rather than total protein abundance [11,13]. In this context, despite increased GRK5 expression, Amlexanox treatment resulted in suppression of NF-kB signaling and inflammatory mediators, indicating functional inhibition of GRK5-dependent pro-inflammatory pathways. Furthermore, the concomitant upregulation GRK5 and MEF2α observed in the MI + AMX group may reflect activation of a protective GRK5/MEF2α transcriptional axis, which has been implicated in restraining myocardial inflammation and adverse remodeling [15,38].

This study has several limitations that should be addressed. Firstly, physiological outcomes of heart functions were not measured, which may be essential for understanding the pathophysiology underlying cardiovascular diseases, and this should be addressed in a future study. An additional limitation is that further investigation regarding the molecular link between GRK5/MEF2 and the NF-ĸB signaling pathway is required. Further studies of its effect using primary cardiomyocytes, for additional confirmation of the underlying molecular mechanism, are suggested. Moreover, studies of the effect of Amlexanox as a cardioprotective agent are limited to preclinical studies; additional studies are needed to assess its efficacy in cardiovascular diseases. Finally, the immunohistochemical and molecular analyses were performed using a relatively small number of animals (n = 3 per group), partly due to limited tissue availability following extensive biochemical and histopathological assessments. Therefore, future studies employing larger sample sizes will be valuable to further validate these mechanistic findings.

## 4. Materials and Methods

### 4.1. Animals

Thirty-two adult BALB/c Albino mice, 8–12 weeks old, weighing 20–25 g, were supplied by The Animal Care Centre at the College of Pharmacy, King Saud University, Riyadh, Saudi Arabia. The mice were kept in optimal conditions, including an air-conditioned room (25 ± 1 °C), a 12 h light/dark cycle, and 60% humidity, and they were fed with tap water ad libitum. The experimental design follows the guidelines of the KSU Experimental Animals Ethics Committee (KSU-SE-23-86).

### 4.2. Induction of Myocardial Infarction

Myocardial infarction was induced using isoproterenol (ISO) (CAS No.: 51-30-9) was purchased from Sigma-Aldrich (St. Louis, MO, USA). ISO was dissolved in normal saline and then injected into mice (100 mg/kg; i.p.) on two consecutive days with an interval of twenty-four hours [39].

### 4.3. Experimental Animal Design

Mice were acclimatized for one week. After that, the animals were randomly divided into four groups, consisting of eight mice each, as follows:

Group 1: Normal control mice received normal saline throughout the experiment.

Group 2: (MI): mice received normal saline for 20 days. Then, they were injected with ISO (100 mg/kg; i.p.) on two constitutive days (days 21 and 22) [39].

Group 3: (MI + V): mice were pre-treated with the vehicle for 20 days. Then, they were injected with ISO (100 mg/kg, i.p.) on two consecutive days (days 21 and 22) [39].

Group 4: (MI + AMX): mice were pre-treated with Amlexanox (2.5 mg/100 g/day IP) for 20 days [30]. Then, they were injected with (100 mg/kg, i.p.) on two constitutive days (days 21 and 22) [39].

At the end of the experiment, all mice were then weighed, anesthetized (by carbon monoxide gas), and euthanized [40]. Trunk blood samples were collected, and the heart was isolated and weighed to obtain the heart weight-to-body weight ratio. Part of the heart tissue was fixed in formalin, while the other part was quickly frozen in liquid nitrogen to be stored at −80 °C for analysis.

### 4.4. Examination of Cardiac Injury and Inflammatory Biomarkers

To determine MI and cardiac injury, serum levels of troponin 1 (Tn-I) and creatine kinase MB (CK-MB) were evaluated using an enzyme-linked immunosorbent assay kit (ELISA) (Solarbio Life Sciences, Tongzhou District, Beijing, China) (Tn-I; cat#SEKR-0048) and (CK-MB; cat#SEKR-0059) according to the manufacturer’s instructions. Moreover, lactate dehydrogenase (LDH) was evaluated using a colorimetric assay kit. Myocardium tissue levels of the TNF-α, IL-6, NF-κB(p65), and NF-κB(p105) subunit were assessed using an ELISA kit from Solarbio Life Sciences, Tongzhou District, Beijing, China. (IL-6; cat# SEKR-0005), (TNF-α; cat#SEKR-0009), (NFKB-p65; cat#SEKR-0168), and (NFKB-p105; cat#SEKR-0148) according to the manufacturer’s instructions. Briefly, an equal weight (200 mg) of mouse cardiac ventricle myocardium tissue (for ≥4 different mice in each group) was homogenized and used to detect target protein levels according to the manufacturer’s instructions. The level of each marker was detected, and the standard curve was created according to the manufacturer’s instructions. Colorimetric results were read utilizing a BioTek Microplate Reader (BioTek Instruments Inc., Winooski, VT, USA) at a specified wavelength. The total amounts of the targets were verified by linear regression analysis and compared with identified concentrations of standards.

### 4.5. Histopathological Analysis

Collected heart samples were cut into pieces and fixed in 10% formalin purchased from Fisher Scientific Ltd. (Loughborough, LE, UK). Samples were dehydrated after fixation using increasing grades of ethanol and then cleared by xylene and infiltrated using molten wax. Samples were embedded in paraffin blocks, which were then sectioned at 7 µm and dried. Sections were stained using hematoxylin and eosin and imaged by light microscopy (Nikon, Tokyo, Japan) [29]. The inflammatory cells were counted using ImageJ software and classified according to the following pathological score: no foci = none, <2 foci per field = mild, 2–4 foci per field = moderate, >4 foci per field = severe [41].

### 4.6. Immunohistochemistry

For immunohistochemical and molecular analyses, three independent animals per group were analyzed. Due to the extensive number of biochemical and histopathological assessments performed on each heart, tissue availability per animal was limited. Therefore, a subset of tissues from randomly selected animals per group was allocated for immunohistochemical and molecular analyses. For each animal, multiple tissue sections and randomly selected microscopic fields were examined to minimize sampling bias.

Heart sections were used to detect the target antigens using paraffin blocks after being treated with 4% paraformaldehyde. All immunohistochemistry reagents used were purchased from Fisher Scientific Ltd. (Bishop Meadow Road, Loughborough, LE, UK). The sections were rehydrated, dewaxed in xylene, and treated with 3% hydrogen peroxide, followed by a 20 min microwave-assisted antigen retrieval procedure. The sections were then washed with PBS and counterstained with MayER’s hematoxylin; then, they were viewed under a microscope after being treated with diaminobenzidine (DAB) purchased from Vector Laboratories, Inc. (Newark, CA, USA) for 5 min to identify immunoreactivity according to the following protocol for the detection of GRK5 and MEF2α antigens.

### 4.7. Reverse Transcription Polymerase Chain Reaction (RT-PCR)

RT-PCR analyses were performed using cardiac tissues obtained from the same randomly selected animals described above (Section 4.6).

The expression of *GRK5*, *MEF2α*, *NF-κB*(*p65*), and *NF-κB* genes was examined in the ventricular myocardium tissue. RNA was extracted using TRIzol reagent (Invitrogen/Life Technologies, Carlsbad, CA, USA) according to the manufacturer’s instructions. RNA samples (1 μg) were reverse transcribed under standard conditions in 20 μL of reaction medium using the Applied Biosystems reverse transcription kit (Invitrogen/Life Technologies, USA). Quantitative RT-PCR amplification of the required genes was made using EverGreen Universal Real-Time PCR Master Mix (Haven Scientific, Makkah, Saudi Arabia). The PCR primer sequences for the target genes are listed in Table 1 and were obtained from Haven Scientific, Saudi Arabia. The reaction conditions were standardized and optimized. The *GAPDH* gene was amplified as an internal control, and gene expression was quantified for each required target gene using the 2^−∆∆Ct^ method [42].

### 4.8. Statistical Analysis

Data are expressed as mean ± SEM. Differences between the groups were determined by one-way ANOVA statistical analysis, followed by an appropriate post hoc test using GraphPad Prism 9. A *p*-value < 0.05 is considered significant.

## 5. Conclusions

**Figure 6:**
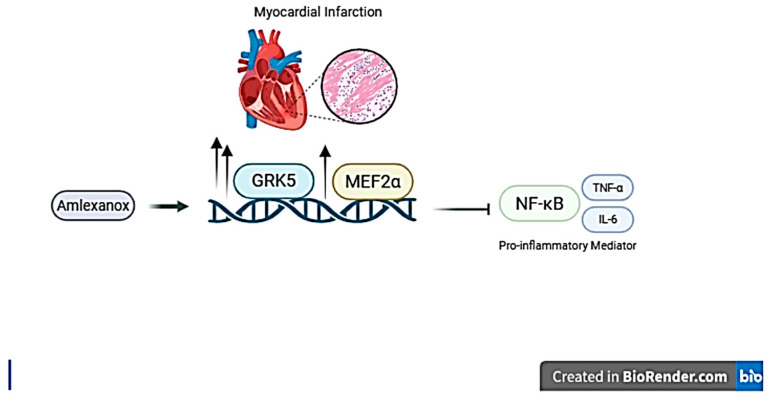
Graphical summary of the Effects of Amlexanox on GRK5-MEF2α-mediated inhibitory effect on NF-κ-B/pro-inflammatory mediator. Created in https://BioRender.com.

In conclusion (Figure 6), our study highlights the promising function of GRK5 in the prevention of MI-provoked cardiac myocyte inflammation. Previous studies have shown the effect of Amlexanox as a GRK5 inhibitor [18]. In addition to that, the transcription factor NF-κB has a central role in cardiac pathology [7]. It has been shown that activation of NF-κB in the myocardium stimulated upregulation of pro-inflammatory cytokines-mediated hypertrophic and fibrotic gene expression. Therefore, the current study established that Amlexanox modulates NF-κB-mediated inflammation, possibly via GRK5/MEF2 upregulation. These findings confirmed the mechanism by which GRK5 exerts its cardioprotective effects. Additionally, our findings implicate GRK5 as a promising therapeutic target for cardiac diseases. Further investigations could be performed using a genetically modified animal model to further confirm the link between GRK5 kinase activity and NF-κB-mediated pro-inflammation in MI.

## Figures and Tables

**Table 1:** Forward and reverse primers oligo sequences (5′-3′) used for RT-PCR.

Gene	Forward	Reverse
*GRK5*	CAAGGAGCTGAATGTGTTCGGAC	GCTGCTTCCAGTGGAGTTTGAAT
*NF-κB*(*p65*)	CAAGTGCCTTAATAGCAGGGCAAA	AGAGCTAGAAAGAGCAAGAGTCCA AT
*NF-κB*	ATGGCAGACGATGATCCCTAC	TGTTGACAGTGGTATTTCTGGTG
*MEF2α*	ACACGCATAATGGATGAGAGGAACCGAC	CAACGATATCCGAGTTCGTCCTGCTTTC
*GAPDH*	GGTTGTCTCCTGCGACTTCA	TGGTCCAGGTTTCTTACTCC

Primer sequences used for RT-PCR [14,43,44]. GRK5, G protein-coupled receptor kinase 5; NF-κB, nuclear factor kappa-light-chain-enhancer of activated B cells; MEF2α, myocyte-specific enhancer factor 2A; GAPDH, glyceraldehyde 3-phosphate dehydrogenase.

## Data Availability

The raw data supporting the conclusions of this article will be made available by the authors on request.

## Abbreviations

The following abbreviations are used in this manuscript:

CK-MB	Creatine kinase MB
ELISA	Enzyme-linked immunosorbent assay
GPCR	G-protein coupled receptor
GRK	G protein-coupled receptor kinase
GRK5	G protein-coupled receptor kinase 5
GAPDH	Glyceraldehyde 3-phosphate dehydrogenase
IκBα:	Nuclear factor of kappa light polypeptide gene enhancer in B-cells inhibitor, alpha
IL-1	Interleukin-1
IL-6	Interleukin-6
ISO	Isoproterenol
IP	intraperitoneal
MI	Myocardial Infarction
MEF2α	Myocyte-specific enhancer factor 2A
NF-κB	Nuclear factor kappa-light-chain-enhancer of activated B cells
Tn-I	Troponin 1
TNFα	Tumor necrosis factor-α
